# Robustifying Vector Median Filter

**DOI:** 10.3390/s110808115

**Published:** 2011-08-18

**Authors:** Samuel Morillas, Valentín Gregori

**Affiliations:** Instituto Universitario de Matemática Pura y Aplicada, Universidad Politécnica de Valencia, Camino de Vera s/n 46022 Valencia, Spain; E-Mail: vgregori@mat.upv.es

**Keywords:** robust filter, color image filter, vector median filter

## Abstract

This paper describes two methods for impulse noise reduction in colour images that outperform the vector median filter from the noise reduction capability point of view. Both methods work by determining first the vector median in a given filtering window. Then, the use of complimentary information from componentwise analysis allows to build robust outputs from more reliable components. The correlation among the colour channels is taken into account in the processing and, as a result, a more robust filter able to process colour images without introducing colour artifacts is obtained. Experimental results show that the images filtered with the proposed method contain less noisy pixels than those obtained through the vector median filter. Objective measures demonstrate the goodness of the achieved improvement.

## 1. Introduction

Image denoising has been for years a very active research topic within image processing because of its necessity for most computer vision systems. In general, The process of denoising or filtering a signal consists of transforming an input signal into another more suitable one for a given purpose. For images, this means to reduce as much as possible the contaminating noise. Several types of noise have been studied in the literature. They may appear alone or be mixed in the digital images. In this work we focus on the impulse noise case, which affect a number of pixels in the image by replacing its original values with other very different ones.

The first denoising methods were linear approaches designed for gray-scale images. Later, it was found that nonlinear methods exhibit better performance and, in particular, when the images are contaminated with impulse noise the median operator [1,2] is the most robust method. Recently, the interest in employing colour images has grown in a wide range of applications, which has motivated the development of colour image filters. The simplest attempts for processing colour images, known as componentwise methods, are based on applying a method for gray-scale images in each of the three colour channels independently [3]. However, it is well known that this way of processing is not appropriate for colour images because there exists a high correlation among the colour image channels which is necessary to be taken into account [3,4]. Otherwise, it may happen that when obtaining the colour for a particular image pixel, components of very different image pixels may be combined, which may generate artificial colours also known as artifacts. This happens, for instance, in the *Vector Marginal Median Filter* (VMMF) [3] that uses the scalar median operator in each of the colour channels. Such a fact implies that componentwise methods are not useful for real applications.

From another point of view, the *Vector Median Filter* (VMF) [5] proposes to process the colour images by treating them as a vector field in order to take into account the interchannel correlation. The family of vector filters inspired by the VMF, which includes the *Directional Vector Filter* [6] among others [3,4,7], is based on the theory of robust statistics [2,8]. These filters perform by selecting as output the vector in a given population which is the closest one to the rest of the vectors in terms of a distance measure. The filters of this family, and specially the VMF, can perform quite robustly in impulse noise reduction without introducing colour artifacts, since they appropriately consider the colour components correlation.

Many approaches have been proposed to improve the performance of these earliest vector filters. In particular, it has been studied how to improve their ability to preserve image details. Since these vector filters apply the filtering operation over all image pixels, many noise free pixels are unnecessarily modified. To solve this problem, there exist mainly two families of methods: on the one hand the so-called switching filters, which are based on a noise detection and replacement approach [9–13] and, on the other hand, filters based on fuzzy logic [14–19], which perform adaptively. Both families are, consequently, able to improve the performance from this point of view. However, the VMF operation is commonly used in many of these recent methods to reduce noise when detected. On the other hand, it is also interesting to note that the noise reduction capability of the VMF can also be improved. For instance, the VMMF exhibits a higher capability to reduce noise. Consider a vector population where all vectors have one noisy component, in this case, the VMF will always select a noisy vector as output. Some recent research has been done with the aim of improving the robustness of VMF. In [20], it is studied how to mix the VMF and the VMMF operations to obtain better noise reduction capability without introducing colour artifacts. In this work we follow this line of research. We introduce two novel methods able to exhibit a more robust performance than the VMF without introducing colour artifacts. The proposed methods are based on replacing some components of the vector median in a given population with other more reliable ones to obtain a more robust filter output. The replacement is based on information extracted from a robustness analysis of the values in each colour channel, and the introduction of colour artifacts is avoided by using a fuzzy similarity based ordering of the vectors with respect to the vector median, which uses fuzzy inference in a similar way to [21]. Experimental results show that the images filtered with the proposed methods contain less noisy pixels than those obtained through the vector median filter. Objective measures demonstrate the goodness of the achieved improvement.

The paper is organized as follows. Section 2 recalls the basics of the VMF. The proposed filtering methods are introduced in Section 3. Experimental results and comparisons are provided in Section 4 and, finally, Section 5 presents the conclusions.

## 2. Basics of Vector Median Filter

Denote by **F** a colour (or multichannel) image to be processed and let *W* be a filtering window centered on the pixel under processing of size *N* × *N*, *N* = 3, 5, 7 . . . containing *N*^2^ = *n* pixels. The colour vectors in *W* are denoted as $F_{j}=(F_{j}^{R},F_{j}^{G},F_{j}^{B}),\;\;\;j=0,1,\ldots ,n-1$. The distance between two vectors **F***_i_*, **F***_j_* is denoted as *ρ*(**F***_i_*, **F***_j_*). In this work, we take the Euclidean distance as the *ρ* function, but any other function fulfilling metric-like properties could be used instead [3,4,12,19,22,23].

The VMF approaches the problem of noise reduction by looking for the most robust vector in the population. For this, each vector in the filtering window is associated to an accumulated distance to all other vectors which is computed as $R_{i}=\sum_{j=0}^{n-1}\rho (F_{i},\;\;F_{j})$. Thus, *R_i_* is the distance associated to the vector **F***_i_*. Then, the colour vectors are ordered according to *R_i_*, so that the ordering of the *R_i_*’s: *R*_(0)_ ≤ *R*_(1)_ ≤ … ≤ *R*_(_*_n_*_−1)_, implies the same ordering of the vectors **F***_i_*’s:

(1) $$F_{\left(0\right)}\leq F_{\left(1\right)}\leq \ldots \leq F_{\left(n-1\right)}$$

Given this order, which is known as reduced ordering of the vectors, the output **VM** is **F**_(0)_, which is the colour vector associated to the minimum accumulated distance. Notice that because of the vector approach, the correlation among the **VM** components is considered, which avoids the generation of colour artifacts. However, in a very noisy context where all colour vectors contain some noisy component, **VM** will be noisy. In the following section we introduce a method intended to increase the noise reduction capability of the **VMF**.

## 3. Proposed Methods

As mentioned above, our proposal is based on taking the vector median **VM** = (*VM^R^*, *VM^G^*, *VM^B^*) and replace some (or all) of its components with other more reliable ones.

In the first step of the proposed method, we perform the reduced ordering operation above over the values of each colour channel independently. Then, the reduced ordering is done over scalar values. For this, we take the absolute value of the scalar difference between the same components of two colour vectors as the distance measure to minimize. Thus, we obtain the following three orderings of values

(2) $$F_{\left(0\right)}^{R}\leq F_{\left(1\right)}^{R}\leq \ldots \leq F_{\left(n-1\right)}^{R}$$

(3) $$F_{\left(0\right)}^{G}\leq F_{\left(1\right)}^{G}\leq \ldots \leq F_{\left(n-1\right)}^{G}$$

(4) $$F_{\left(0\right)}^{B}\leq F_{\left(1\right)}^{B}\leq \ldots \leq F_{\left(n-1\right)}^{B}$$

Which are associated to the R, G and B colour channels, respectively, and we say, for instance, that the rank in (2) of $F_{i}^{R}$ is *r* if $F_{i}^{R}=F_{\left(r\right)}^{R}$. Notice that this ordering provides information about the robustness of each vector component so that the most robust components occupy the lower ranks. Also, the ordering obtained is the same to the ordering made in the VMMF, which takes as output in each channel the value in the lowest rank.

Second, we order the colour vectors in *W* according to their similarity with respect to the **VM**. Usually, the similarity between two colour vectors is measured using the differences in the three components. For instance, when using the Euclidean distance, other metrics or fuzzy metrics [12]. However, our method is designed to operate in a very noisy context, where in some cases all pixels in a window may have one noisy component. So, we relax this condition by considering that two colour vectors are similar if two of their components are similar. This allows a vector with a noisy component to be considered similar to another. We define this similarity by means of the following fuzzy rule

**Fuzzy Rule 1** *Computing the similarity between* **F***_i_* *and* **F***_j_*:

if $\left|F_{i}^{R}-F_{j}^{R}\right|$ *is* small *AND* $\left|F_{i}^{G}-F_{j}^{G}\right|$ *is* small
  *OR*
  
$\left|F_{i}^{G}-F_{j}^{G}\right|$ *is* small *AND* $\left|F_{i}^{B}-F_{j}^{B}\right|$ *is* small
  *OR*
  
$\left|F_{i}^{R}-F_{j}^{R}\right|$ *is* small *AND* $\left|F_{i}^{B}-F_{j}^{B}\right|$ *is* small
then **F***_i_* *and* **F***_j_* *are similar*.

The certainty degree of the vague statement in the consequence of the fuzzy rule is obtained through fuzzy inference as follows. The interested reader can find extensive information on the fuzzy inference process in the literature, for instance in [24]. To assign the certainty degree of the vague statement “ $\left|F_{i}^{R}-F_{j}^{R}\right|$ is *small*” we use a function [25] *μ* given by $\mu (\left|F_{i}^{R}-F_{j}^{R}\right|)=1-\frac{\left|F_{i}^{R}-F_{j}^{R}\right|}{K}$, where *K* = 255 is the maximum value that can take the difference $\left|F_{i}^{R}-F_{j}^{R}\right|$ (actually, *μ* can be considered as a fuzzy metric [25] $M(F_{i}^{R},\;\;F_{j}^{R})=1-\frac{\left|F_{i}^{R}-F_{j}^{R}\right|}{K}$). Analogously and using the same *μ* function, we assign the rest of the certainties of vague statements in the antecedent of Fuzzy Rule 1. Now, for computing the certainty degree of the antecedent of Fuzzy Rule 1, following the usual procedure in fuzzy logic, we apply the conjunction operation AND and the disjunction operation OR by means of a t-norm * and its associated s-norm *′, respectively. In this paper, we use the usual product as the t-norm and the probabilistic addition as the s-norm. This certainty is identified with the degree to which **F***_i_* and **F***_j_* are similar, denoted by *s*(**F***_i_*, **F***_j_*), so that

$$\begin{array}{ll} s(F_{i},\;\;F_{j}) & =(\mu (\left|F_{i}^{R}-F_{j}^{R}\right|)*\mu (\left|F_{i}^{G}-F_{j}^{G}\right|)){*}^{\prime }(\mu (\left|F_{i}^{G}-F_{j}^{G}\right|)\\ & *\mu (\left|F_{i}^{B}-F_{j}^{B}\right|)){*}^{\prime }(\mu (\left|F_{i}^{R}-F_{j}^{R}\right|)*\mu (\left|F_{i}^{B}-F_{j}^{B}\right|)) \end{array}$$

Third, by ordering the colour vectors according to their similarity with **VM** we obtain the ordered set

(5) $$W^{\prime }=\{F_{\left[0\right]},\;\;F_{\left[1\right]},\;\ldots \;,F_{\left[n-1\right]}\}$$

such that

(6) $$s(\mathrm{VM},\;\;F_{\left[0\right]})\geq s(\mathrm{VM},\;\;F_{\left[1\right]})\geq \ldots \geq s(\mathrm{VM},\;\;F_{\left[n-1\right]})$$

where **F**_[0]_ = **VM**. Now, consider that we take a low value *t*, 1 ≤ *t* < *n*, and we take a subset *S_t_* of *W*’ containing the *t* most similar colour vectors to **VM**:

(7) $$S_{t}=\{F_{\left[0\right]},F_{\left[1\right]},\ldots ,F_{\left[t-1\right]}\}$$

If *t* is low enough, *t* << *n*, *S_t_* contains a set of colour vectors that may have some noisy components but that also have similar components to **VM**.

Finally, from the previous information, we will determine a colour vector more robust than **VM**. For this, we propose two different options which result in two different filtering methods.

### 3.1. Selective Replacement of Components of **VM**

**VM** occupies the lowest rank in the ordering (1) and it would be expected that its components have low ranks in the orderings (2)–(4). However, as commented above, **VM** may have some noisy components in very noisy situations. Therefore, we propose to detect components of **VM** that may be noisy using the information in the orderings (2)–(4) and replace them with components of vectors in *S_t_* that have a low rank in (2)–(4). Notice that this replacement can be safely done without introducing colour artifacts since *S_t_* only contains colour vectors with components similar to those of **VM**, which can be combined safely. Moreover, the component of **VM** will not be replaced with another noisy component since we only use components with low ranks in (2)–(4).

Specifically, in the following we explain in detail how to make the mentioned replacements obtaining the *Modified Vector Median* (**MVM**). We detail the procedure for the R channel component but we process analogously the G and B channels.

Let us denote by *r* the rank of *VM^R^* in (2). We will keep the original component *VM^R^* if *r* ≤ *t* − 1 or *r* is lower than the ranks of the components $F_{\left[1\right]}^{R},\ldots ,F_{\left[t-1\right]}^{R}$. Otherwise, we replace *VM^R^* with the component in $\left\{F_{\left[1\right]}^{R},\ldots ,F_{\left[t-1\right]}^{R}\right\}$ with the lowest rank in (2).

### 3.2. Obtaining the Most Robust Colour Vector from the Components in S_t_

Our second proposal is based on obtaining the most robust colour vector using the components in *S_t_*. For this, we take into account the orderings (2)–(4) of the components in *S_t_*. We build the *Robust Vector Median* (**RVM**) taking for each colour channel the component in *S_t_* that occupies the lowest rank in (2)–(4). That is, for the R component, we take the component in $\left\{F_{\left[0\right]}^{R},\ldots ,F_{\left[t-1\right]}^{R}\right\}$ with the lowest rank in (2). For the G and B components we perform analogously using the orderings (3) and (4), respectively. In this way, we combine noise-free components from different colour vectors in *S_t_* but notice that no colour artifacts are obtained since the components of the colour vectors in *S_t_* are similar.

## 4. Experimental Results and Assessment

**Figure 1.**
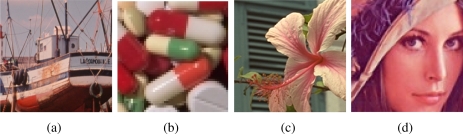
Test images : **(a)** Boats; **(b)** Pills; **(c)** Flower; and **(d)** Lenna.

Impulsive noise corruption process affects only some pixels in the image while leaving other pixels unchanged. Typically, the noise process changes one or more color components of the affected pixel by replacing its original values with other values which usually significantly deviates from the originals. The kind of noise which is the most difficult to detect and remove assumes that the impulse is a random uniformly distributed value within the signal range. For RGB images, we consider that the noise is independently introduced in each of the three colour channels with probability *p*, which means that the probability of a colour vector to have some noisy component is 3*p* − 3*p*^2^ + *p*^3^ (or equivalently, that the (3*p* − 3*p*^2^ + *p*^3^)100% image pixels are corrupted with noise). This noise model has been used to contaminate the test images in Figure 1.

The contaminated images have been filtered using the VMMF, VMF, the recent AMMF [20], and the proposed methods called *Modified Vector Median Filter* (MVMF) and *Robust Vector Median Filter* (RVMF). In all cases we have used a 3 × 3 filtering window and we have filtered each image only once to better appreciate the performance differences. However, when the noise is high, several filtering processes are frequently necessary to obtain a totally clean image. In the case of the AMMF we have set the *m* parameter to *m* = 3, as suggested in [20]. For MVMF and RVMF we have set *t* = 3 because this is the smallest value that makes sense to use and because increasing this value will also decrease the reliability of the method from the artifact generation point of view.

In addition to visual comparison, to assess the quality of the different filters we have used the objective quality measures *Mean Absolute Error* (MAE), *Peak Signal to Noise Ratio* (PSNR), and *Normalized Color Difference* (NCD). The definition of these objective quality measures can be found in [3,4].

**Figure 2.**
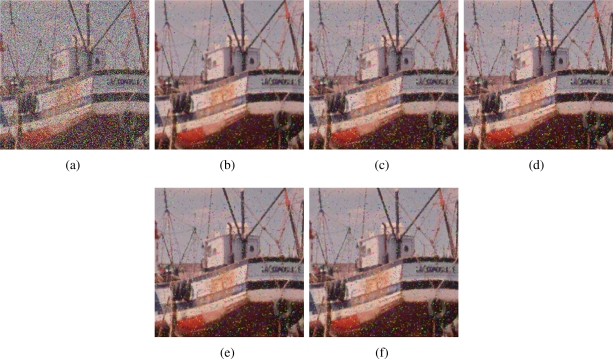
**(a)** Boats image corrupted with random-value impulse noise with *p* = 0.4 in each colour channel and outputs from; **(b)** VMMF; **(c)** VMF; **(d)** AMMF; **(e)** MVMF; and **(f)** RVMF.

**Figure 3.**
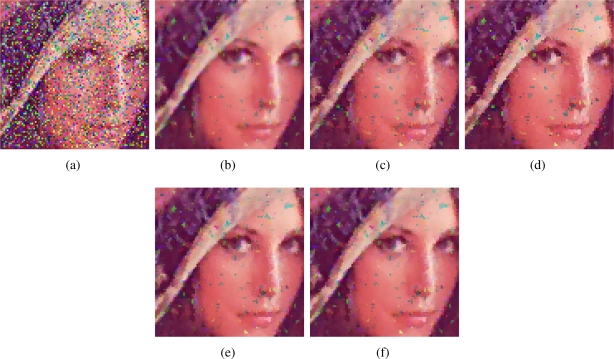
**(a)** Lenna image corrupted with random-value impulse noise with *p* = 0.3 in each colour channel and outputs from; **(b)** VMMF; **(c)** VMF; **(d)** AMMF; **(e)** MVMF; and **(f)** RVMF.

**Figure 4.**
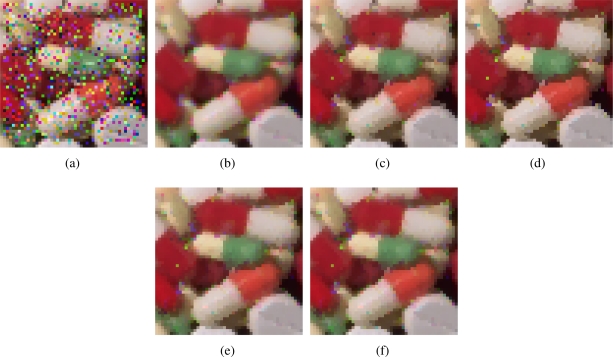
**(a)** Pills image corrupted with random-value impulse noise with *p* = 0.2 in each colour channel and outputs from; **(b)** VMMF; **(c)** VMF; **(d)** AMMF; **(e)** MVMF; and **(f)** RVMF.

**Figure 5.**
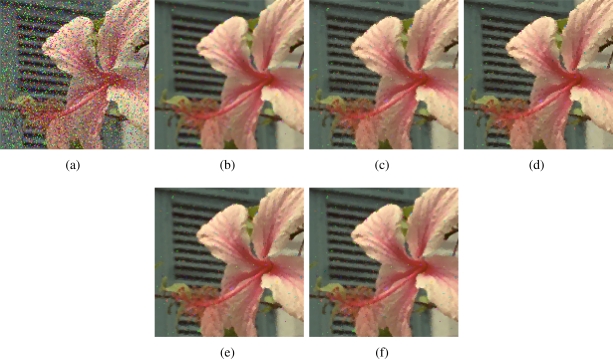
**(a)** Flower image corrupted with random-value impulse noise with *p* = 0.2 in each colour channel and outputs from; **(b)** VMMF; **(c)** VMF; **(d)** AMMF; **(e)** MVMF; and **(f)** RVMF.

**Figure 6.**
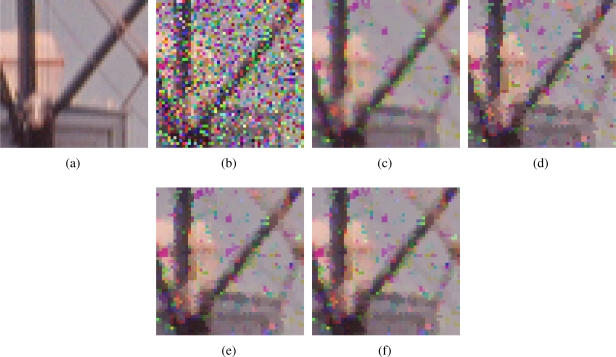
Comparison of colour artifacts on small image patches: **(a)** Small patch of Boats image; **(b)** corrupted with random-value impulse noise with *p* = 0.4 in each colour channel and outputs from; **(c)** VMMF; **(d)** VMF; **(e)** MVMF; and **(f)** RVMF.

**Figure 7.**
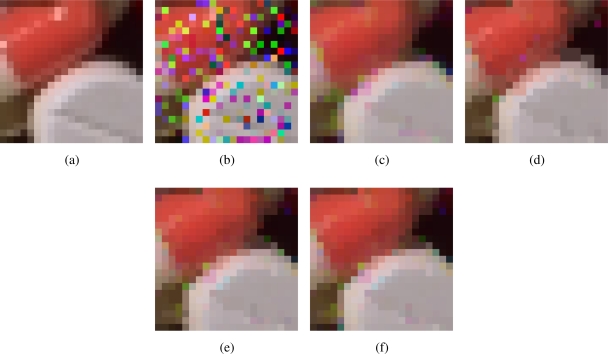
Comparison of colour artifacts on small image patches: **(a)** Small patch of Pills image; **(b)** corrupted with random-value impulse noise with *p* = 0.2 in each colour channel and outputs from; **(c)** VMMF; **(d)** VMF; **(e)** MVMF; and **(f)** RVMF.

Figures 2–7 show some noisy images and the respective filtering results using the methods in the comparison. We can see that the highest noise reduction ability is exhibited by the VMMF, however, this filter also introduces many colour artifacts near edges. This can be seen for instance in dark zones edges in Figure 2(b), near white areas in Figure 4(b) and in Figures 6–7 where we show the performance on two small patches of Boats and Pills image. Clearly, VMMF introduces much more artifact colours than VMF, MVMF and RVMF. The second best noise reduction capability is obtained by MVMF and RVMF, which perform better than AMMF and VMF especially when the noise is high, which is when MVMF and RVMF clearly outperform VMF and AMMF for their increased robustness. The images filtered with MVMF and RVMF contain less noisy pixels than those filtered with the VMF and AMMF and no colour artifacts are introduced by any of these methods.

Numerical results in Tables 1–4 support these facts. The best results are obtained by VMMF followed by RVMF and MVMF. However, we need to take into account that these measures do not reflect specifically the introduction of colour artifacts. This means that, from the numerical point of view, and especially for MAE and PSNR, it is better to reduce more noise even if a few colour artifacts are included. This is why VMMF obtains the best results but, for the introduction of colour artifacts, cannot be used in practical applications. With respect to AMMF and VMF, we can see that numerical results are in favor of RVMF and MVMF and that RVMF performs slightly better than MVMF. In addition, the advantage of RVMF and MVMF with respect to VMMF is that they avoid the introduction of colour artifacts as shown in Figures 6–7.

## 5. Conclusions

In this paper we have introduced two novel methods for impulse noise reduction in colour images able to outperform the classical vector median filter from the noise reduction capability point of view. The proposed methods use the vector median of each filtering window and complimentary information from componentwise analysis to build more robust outputs. Experimental results show that the images filtered with the proposed methods contain less noisy pixels than those obtained through the vector median filter and no colour artifacts are introduced because the correlation among the colour image channels is appropriately taken into account. Objective measures demonstrate the goodness of the achieved improvement. The proposed methods can be included within more complex filtering procedures to process the most noisy regions of the images or to reduce impulse noise when detected, where the vector median filter has been commonly used to date.

## Figures and Tables

**Table 1.** Performance comparison in terms of MAE, PSNR, and NCD when filtering the Boats image contaminated with random-value impulse noise with probability *p* in each colour channel.

**Filter**	*p* = 0.10			*p* = 0.20			*p* = 0.30			*p* = 0.40		
	**MAE**	**PSNR**	**NCD**	**MAE**	**PSNR**	**NCD**	**MAE**	**PSNR**	**NCD**	**MAE**	**PSNR**	**NCD**

None	7.55	18.82	12.02	15.01	15.83	22.60	22.70	14.03	32.28	30.26	12.77	40.40
VMMF	4.30	29.99	3.74	5.24	28.07	5.06	6.78	25.55	7.31	9.52	22.56	11.13
VMF	4.67	29.51	3.19	6.18	26.79	4.72	8.82	23.44	8.29	12.93	20.32	13.76
AMMF	4.73	29.09	3.28	6.01	26.53	4.58	8.18	23.24	7.75	11.86	20.08	13.13
MVMF	4.50	29.70	3.33	5.62	27.40	4.68	7.47	24.45	7.39	10.59	21.36	11.88
RVMF	4.42	29.79	3.50	5.51	27.51	4.81	7.29	24.60	7.43	10.34	21.52	11.80

**Table 2.** Performance comparison in terms of MAE, PSNR, and NCD when filtering the Lenna image contaminated with random-value impulse noise with probability *p* in each colour channel.

**Filter**	*p* = 0.10			*p* = 0.20			*p* = 0.30			*p* = 0.40		
	**MAE**	**PSNR**	**NCD**	**MAE**	**PSNR**	**NCD**	**MAE**	**PSNR**	**NCD**	**MAE**	**PSNR**	**NCD**

None	7.63	18.52	11.33	15.19	15.56	21.37	22.94	13.75	30.20	30.31	12.61	37.79
VMMF	4.85	28.58	4.17	6.06	26.72	5.84	7.97	24.26	8.38	10.70	21.86	11.67
VMF	5.25	28.05	3.45	7.15	25.60	5.18	10.05	22.50	8.68	14.20	19.82	13.54
AMMF	5.44	27.57	3.66	7.04	25.36	5.16	9.36	22.32	8.23	12.98	19.64	12.83
MVMF	5.05	28.30	3.63	6.46	26.28	5.24	8.54	23.44	8.06	11.84	20.76	12.15
RVMF	4.97	28.37	3.85	6.30	26.44	5.42	8.40	23.51	8.24	11.57	20.92	12.11

**Table 3.** Performance comparison in terms of MAE, PSNR, and NCD when filtering the Pills image contaminated with random-value impulse noise with probability *p* in each colour channel.

**Filter**	*p* = 0.10			*p* = 0.20			*p* = 0.30			*p* = 0.40		
	**MAE**	**PSNR**	**NCD**	**MAE**	**PSNR**	**NCD**	**MAE**	**PSNR**	**NCD**	**MAE**	**PSNR**	**NCD**

None	7.46	18.66	11.89	14.72	15.64	22.71	21.94	13.85	30.92	29.56	12.56	38.96
VMM	5.35	26.98	4.91	7.01	25.30	7.23	9.71	22.65	10.01	13.04	20.77	14.26
VMF	5.97	26.55	3.84	8.21	24.30	5.93	12.17	21.17	10.10	16.24	19.29	15.62
AMMF	6.27	25.95	4.19	8.30	23.70	6.24	11.47	20.92	9.82	14.95	19.17	15.10
MVMF	5.66	26.79	4.08	7.45	24.83	6.21	10.58	21.94	9.76	13.92	20.07	14.46
RVMF	5.58	26.83	4.52	7.26	24.96	6.62	10.23	22.17	9.79	13.61	20.18	14.41

**Table 4.** Performance comparison in terms of MAE, PSNR, and NCD when filtering the Flower image contaminated with random-value impulse noise with probability *p* in each colour channel.

**Filter**	*p* = 0.10			*p* = 0.20			*p* = 0.30			*p* = 0.40		
	**MAE**	**PSNR**	**NCD**	**MAE**	**PSNR**	**NCD**	**MAE**	**PSNR**	**NCD**	**MAE**	**PSNR**	**NCD**

None	7.46	18.82	11.57	14.70	15.88	21.63	22.33	14.08	30.87	29.70	12.86	38.77
VMMF	5.00	28.63	3.89	6.25	26.81	5.72	8.06	24.56	7.90	10.87	22.05	11.00
VMF	5.42	28.17	2.97	7.46	25.63	4.54	10.19	22.84	7.71	14.20	20.14	12.49
AMMF	5.56	27.76	3.14	7.38	25.38	4.65	9.53	22.73	7.61	13.10	19.98	12.51
MVMF	5.25	28.40	3.20	6.82	26.22	4.82	8.87	23.70	7.58	12.02	21.04	11.53
RVMF	5.17	28.47	3.47	6.66	26.35	5.08	8.60	23.90	7.66	11.71	21.20	11.51
